# Neural basis of lower-limb visual feedback therapy: an EEG study in healthy subjects

**DOI:** 10.1186/s12984-024-01408-8

**Published:** 2024-07-08

**Authors:** Ahmed Adham, Ba Thien Le, Julien Bonnal, Hugo Bessaguet, Etienne Ojardias, Pascal Giraux, Pascal Auzou

**Affiliations:** 1 Department of Physical Rehabilitation, CHU of St Etienne, Saint-Étienne, France; 2 Laboratory Trajectoires, INSERM 1028, CNRS 5229, University of Lyon-St-Etienne, Saint-Étienne, France; 3 Department of Neurology, CHU of Orleans, Orleans, France; 4 Jean Monnet University, Lyon 1, Université Savoie Mont-Blanc, “Laboratoire Inter-Universitaire de Biologie de La Motricité”, 42023 Saint-Étienne, France; 5 “Laboratoire Interdisciplinaire d’innovation et de Recherche en Santé d’Orléans”, LI2RSO, University of Orleans, Orleans, France; 6 Univ. Grenoble Alpes, CEA, LETI, Clinatec, Grenoble, France

**Keywords:** Video feedback therapy, Lower limb, EEG, Rehabilitation, Mirror neuron system

## Abstract

**Background:**

Video-feedback observational therapy (VOT) is an intensive rehabilitation technique based on movement repetition and visualization that has shown benefits for motor rehabilitation of the upper and lower limbs. Despite an increase in recent literature on the neurophysiological effects of VOT in the upper limb, there is little knowledge about the cortical effects of visual feedback therapies when applied to the lower limbs. The aim of our study was to better understand the neurophysiological effects of VOT. Thus, we identified and compared the EEG biomarkers of healthy subjects undergoing lower limb VOT during three tasks: passive observation, observation and motor imagery, observation and motor execution.

**Methods:**

We recruited 38 healthy volunteers and monitored their EEG activity while they performed a right ankle dorsiflexion task in the VOT. Three graded motor tasks associated with action observation were tested: action observation alone (O), motor imagery with action observation (OI), and motor execution synchronized with action observation (OM). The alpha and beta event-related desynchronization (ERD) and event-related synchronization (or beta rebound, ERS) rhythms were used as biomarkers of cortical activation and compared between conditions with a permutation test. Changes in connectivity during the task were computed with phase locking value (PLV).

**Results:**

During the task, in the alpha band, the ERD was comparable between O and OI activities across the precentral, central and parietal electrodes. OM involved the same regions but had greater ERD over the central electrodes. In the beta band, there was a gradation of ERD intensity in O, OI and OM over central electrodes. After the task, the ERS changes were weak during the O task but were strong during the OI and OM (Cz) tasks, with no differences between OI and OM.

**Conclusion:**

Alpha band ERD results demonstrated the recruitment of mirror neurons during lower limb VOT due to visual feedback. Beta band ERD reflects strong recruitment of the sensorimotor cortex evoked by motor imagery and action execution. These results also emphasize the need for an active motor task, either motor imagery or motor execution task during VOT, to elicit a post-task ERS, which is absent during passive observation.

*Trial Registration* NCT05743647

**Supplementary Information:**

The online version contains supplementary material available at 10.1186/s12984-024-01408-8.

## Introduction

In recent years, innovative new techniques have emerged in the field of neurological rehabilitation. Among these techniques, visual feedback therapies distinguish themselves by their ease of use, low cost and efficacy. The aim of visual feedback therapies is to provide visual feedback of a movement correctly performed by the affected limb to elicit cortical activation.

Historically, mirror therapy was the first rehabilitation technique used to offer patients subjective visual feedback of correct movement performed by a paretic limb [1, 2]. In lower-limb mirror therapy, a mirror is placed between the subject’s legs; the subjects are invited to perform a movement with their healthy leg while observing the mirror’s reflection, which gives them the subjective illusion of moving their paretic leg. During the last decade, Video Observational Therapy (VOT) and Virtual Reality have emerged as alternatives to mirror therapy*.* In these therapies, the subject observes on a screen or in a virtual reality headset a projection of the paretic lower limb performing the action. This projection can be made by using a pre-recorded video of the healthy limb performing the action flipped on the horizontal axis (mirror image) in video observational therapy or by using a robotic avatar in virtual reality. According to multiple meta-analyses, lower limb visual feedback techniques (mirror therapy, VOT, virtual reality) have been shown to improve lower limb function in stroke patients. Mirror therapy has proven to be effective at improving mobility, motor recovery, balance, spasticity, step length and walking speed in stroke patients [3–5], hemineglect [6] and pain [7]. Video feedback therapies (VOT and virtual reality) have shown improvements in dynamic and static balance [8] and in the composite criterion of mobility (10-m walk test, time up and go, functional ambulation category) [9].

Neurophysiological studies carried out on the upper limb provide us with a better understanding of the effects of these therapies. Observing the action during mirror therapy reduces beta rhythms in sensorimotor regions, indicating a rebalancing of the interhemispheric balance [10, 11]. These results were also found for video observational therapy on the upper limb [12]. This motor facilitation is associated with an increase in cortico-spinal excitability in the mirror therapy of the upper limb [13]. This stimulation of sensory-motor regions is achieved through recruitment of the mirror neuron system [14]. There are also changes in cortico-cortical connectivity, particularly between motor areas, the posterior cingulate cortex, the precuneus and visual areas, linked to visuospatial attentional recruitment [15]. For the lower limb, however, we have little physiological data. Mirrored visual feedback leads to recruitment of the ipsilesional sensory-motor cortex during ankle dorsiflexion movement on fMRI [16]. There is also a reorganization of fMRI functional connectivity within the sensorimotor cortex during passive action observation with mirrored visual feedback [17]. However, there is little information on the modulations of EEG rhythms (desynchronizations, beta rebounds, functional connectivity) induced by visual feedback rehabilitation of the lower limb, especially via video feedback techniques. As the healthy limb remains immobile in VOT, this therapy is also a better model than mirror therapy for specifically studying the brain dynamics induced by visual feedback since it allows us to study the effects of pure visual feedback and motor intention of the trained limb, uncontaminated by the cortical activity induced by healthy limb movement in mirror therapy. Furthermore, VOT gives the subjects visual feedback of their own limb movement (appearance, etc.), thus maximizing the embodiment of the therapy, which is less common in virtual reality with a robotic avatar.

Interestingly, a multitude of ways in which the patient can work on these visual feedback therapies exist for rehabilitation. The subject could simply observe the visual feedback passively (simple observation, O). They could also observe it while attempting to produce motor imagery of the movement (motor imagery, OI). Finally, the subject could observe visual feedback while attempting to reproduce the movement (motor execution, OM) [18]. These task differences are significant from a physiological point of view. Indeed, in the upper limb, under conditions similar to first-person VOT, EEG differences have been shown between execution and motor imagery [19]. Similarly, in fMRI, the gradation of engagement in action (OI/OM) is associated with increased activation of sensory-motor areas [20]. Despite these results for the upper limb, we have few points of comparison in the literature on the neurophysiological differences between the O, OI and OM conditions for lower limb tasks in rehabilitation.

The aim of this study was to explore the EEG correlates of video feedback therapy. To this end, we studied a cohort of 38 healthy subjects who passively observed (O) or observed while imagining (OI) or observed while performing (OM) a dorsiflexion movement of their right ankle while performing computerized first-person video observational therapy. EEG Biomarkers in the alpha and beta bands (Event related desynchronisation and Event related synchronisation) were studied and compared between conditions and between groups.

Event related desynchronisation (ERD) is defined as a decrease of power in the alpha and beta band during the movement, while the Event related synchronization (ERS or beta rebound) refers to a post-movement synchronization period. ERD and ERS rhythms are generally observed above the motor regions contralateral to the limb producing the movement. More specifically, alpha desynchronization is generally known to reflect recruitment of mirror neurons [21] and to support somato-sensory rhythms [22, 23], while beta desynchronization is also often associated with the recruitment of motor cortex [23, 24]. Beta rebound, on the other hand, is associated with post-movement motor validation phenomena [25, 26]. Therefore, as the subjects observe in all conditions (O, OI, OM) the visual feedback of their moving limb, we hypothesized a systematic recruitment of the mirror neurons system, leading to an alpha band desynchronization over sensori-motor regions in all conditions. We also hypothesized a stronger recruitment of sensorimotor cortex in OI task as compared to O, and in OM task as compared to O and OI, due to the addition of motor imagery and motor execution to action observation. This would be reflected by a gradation in the ERD strength (OM_ERD_ > OI_ERD_ > O_ERD_). We expected the same dynamics for beta ERS.

Functional connectivity was also studied between conditions, to identify and describe network organization changes over time for lower limb movements. Having few points of comparison in the literature, we had no assumptions about the results.

## Materials and methods

### Participants

Thirty-eight healthy volunteers aged between 20 and 73 y.o. (age: 45.5 y.o. ± 20 y.o.) participated in the study. There were 26 males and 12 females, 25 subjects aged younger than 60 y.o. and 13 subjects aged older than 60 y.o. Prior to the recording, the subjects’ handedness was assessed with the Edinburgh Handedness Inventory [27]. Patients who presented with neurological disease or psychiatric illness or who were receiving neuro-modulatory treatments were excluded from our study. Participants signed a consent form prior to participating in the study. The study was conducted in accordance with the Declaration of Helsinki and was approved by the ethics committee “Comité de protection des personnes Sud-Est III” (2022-A02375-38) and registered in Clinical Trials (NCT05743647).

### Material

**Fig. 1:**
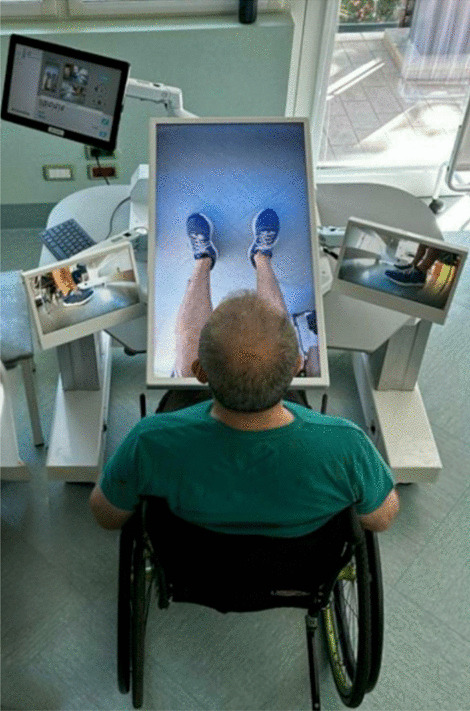
First-person lower-limb video feedback therapy—IVS4—DESSINTEY

Participants were comfortably seated in a standardized (hip knees and ankles flexed at a 90° angle) position on a chair in front of a height-adjustable table on which they trained on VOT. We used the IVS4™ (Dessintey Co., France) device (Fig. 1), which uses a large screen precisely placed between the subject’s eye and the trained hand. The device is equipped with a camera placed in front of the legs. The camera recorded the left lower limb movement of the subject. The recorded videos were mirrored and later projected on a screen, giving the subject a visual illusion that the movement was performed by the right limb. The advantage of this device is that it can easily provide first-person feedback congruent with the visual axis, thus maximizing subjective illusions. It is to be noted that the subjects didn’t directly perform a right foot movement, despite being healthy controls, since the VOT device automatically mirrors the image.

EEG data were recorded with a 32-channel ENOBIO™ device (Neuroelectrics Co., Spain) placed in a standard position on the head of the subjects with Ag/AgCl electrodes. The data were sampled at 500 Hz, and the impedance was maintained below 5 kHz. The protocol displayed on the IVS panel was designed with Open-Sesame software [28]. An Open-Sesame TTL trigger was sent on the EEG recording at the beginning of each experimental condition for precise synchronization of the visual cues in further analysis.

### Experimental device

**Fig. 2:**
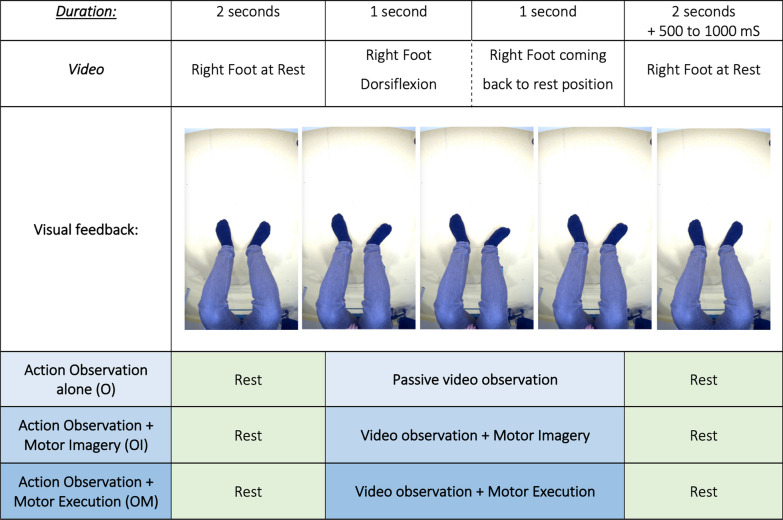
Experimental paradigm for O (Action Observation alone), OI (Action Observation + Motor Imagery) and OM (Action Observation + Motor Execution M) conditions

Subjects underwent a single EEG recording session. Prior to the experiment, we recorded a video of the subject’s left foot performing a movement of ankle dorsiflexion. The video was then manually extracted from the VOT device, mirrored, cut and resampled in Adobe Premiere Pro for the whole movement to last exactly two seconds, with two seconds of pause before and after the movement. The movement displayed and performed by the subjects via both techniques consisted of smooth dorsiflexion of the right foot (one second) immediately followed by flexion of the foot (one second). At rest, the foot was on the ground and completely relaxed. The timing of the whole video was as follows: two seconds of presentation of the right foot at rest, two seconds of task, and two seconds of rest (Fig. 2). We added a randomized time of 500 to 1000 ms at the end of each 6-s video.

Each session was divided into three sub-sessions, separated by a one-minute pause. In each sub-session, the subject performed thirty movements: (1) action observation alone, (2) action observation + motor imagination, and (3) action observation + motor execution. The order of the sub-sessions was randomized between subjects.

### Data analysis

#### Time–frequency analysis

After filtering (0.5–70 Hz bandpass filter, 50 Hz notch filter), the data were segmented into 6-s epochs. The epochs containing a peak-to-peak voltage above 100 mV were considered too noisy and rejected. Then, a visual inspection of the data was conducted while rejecting the remaining noisy epochs, and bad channels were interpolated. Approximately 80% of the data in our study were considered valid. The data were referenced to an infinite source with the REST algorithm [29]. Ocular artifacts were removed via independent component analysis (ICA). This whole process was conducted with the MATLAB EEGLab Toolbox (UC San Diego, USA, 30). After this pre-treatment, for each epoch and each EEG channel, time–frequency maps were generated. We implemented time–frequency analysis by convolving the signal with a set of complex Morlet wavelets, defined as complex sine waves tapered by a Gaussian function. The frequencies of the wavelets ranged from 2 to 40 Hz in 80 linearly spaced steps. The full width at half maximum (FWHM) ranged from 1000 to 200 ms [31, 32].

For each electrode, ERD (event-related desynchronization) and ERS (event-related synchronization) magnitudes were then expressed as a percentage of the power in the defined time and frequency window relative to the power measured during the corresponding baseline [33] and were expressed as a percentage change. The baseline was chosen between 1500 and 500 ms before the onset of the movement. We analyzed (i) alpha band power during the task (2500–3500 ms) to determine the alpha component of the Mu motor rhythm (Alpha ERD), (ii) beta band power during the task (2500–3500 ms) to obtain the beta component of the Mu motor rhythm (Beta ERD), and (iii) beta band power after the task (4000–5000 ms) to obtain the post-movement beta rebound power (ERS). We chose to remove the first and last 500ms around the task in the time–frequency analysis since during the motor execution condition (OM) the subjects weren’t always perfectly synchronized with the video in the beginning or ending of the movement: some subjects started or ended the movement a little earlier or later, which may have confused statistical analysis.

The alpha and beta central frequency bands were adaptively adapted to each subject: the mean and full-width at half-maximum of the alpha and beta spectral distributions were defined for each subject as described in Stolk et al. [23] using a two- or three-way Gaussian model depending on the presence or absence of slow theta-delta waves. On average, the alpha band central frequency was 9.3 ± 1.3 Hz, and the beta band central frequency was 16.2 ± 1.7 Hz. This adaptive central frequency choice diminished the overlaps between signals in the alpha and beta bands that can occur with a canonical choice of frequency bands.

#### Connectivity analysis

Connectivity analyses were performed before and during the task for each subject. First, the broadband time-domain source signals were bandpass filtered in the alpha or beta central frequency for each subject. A space Laplacian filter was applied to the data to minimize the effects of volume conduction [34]. A Hilbert transform was then applied to the data before assessing connectivity with the phase locking value (PLV). The PLV is a normalized value that gives for each pair of electrodes a value ranging from 0 (no phase locking) to 1 (complete phase locking) and is defined by Eq. (1), from [35], where n indexes the trial number and N is the total number of trials.

Equation 1: Phase locking value formula, from Aydore et al.

1 $${\widehat {PLV}_{sample}} \triangleq \left| {\frac{1}{N}\sum\limits_{n = 1}^N {{e^{j\Delta {\varphi_n}(t)}}} } \right|$$

For each electrode, we computed the connectivity strength, defined as the sum of weights of links connected to the node. We then subtracted the connectivity strength map during the movement from the connectivity strength map before the movement to visualize how connectivity changed during and after the movement compared to the baseline. We also plotted maps of the individual links that increased by more than 2 standard deviations compared with the average change in the PLV.

### Statistical comparison

For the time–frequency and connectivity statistical comparisons, we compared the different conditions with a nonparametric permutation test (10.000 permutations, p < 0.025, 36). Multiple comparison correction was performed with a Holm–Bonferroni correction. To ease the visualization of the data, we plotted cortical maps showing the power modifications expressed as percentages of change only in regions with suprathreshold significant differences.

## Results

**Fig. 3:**
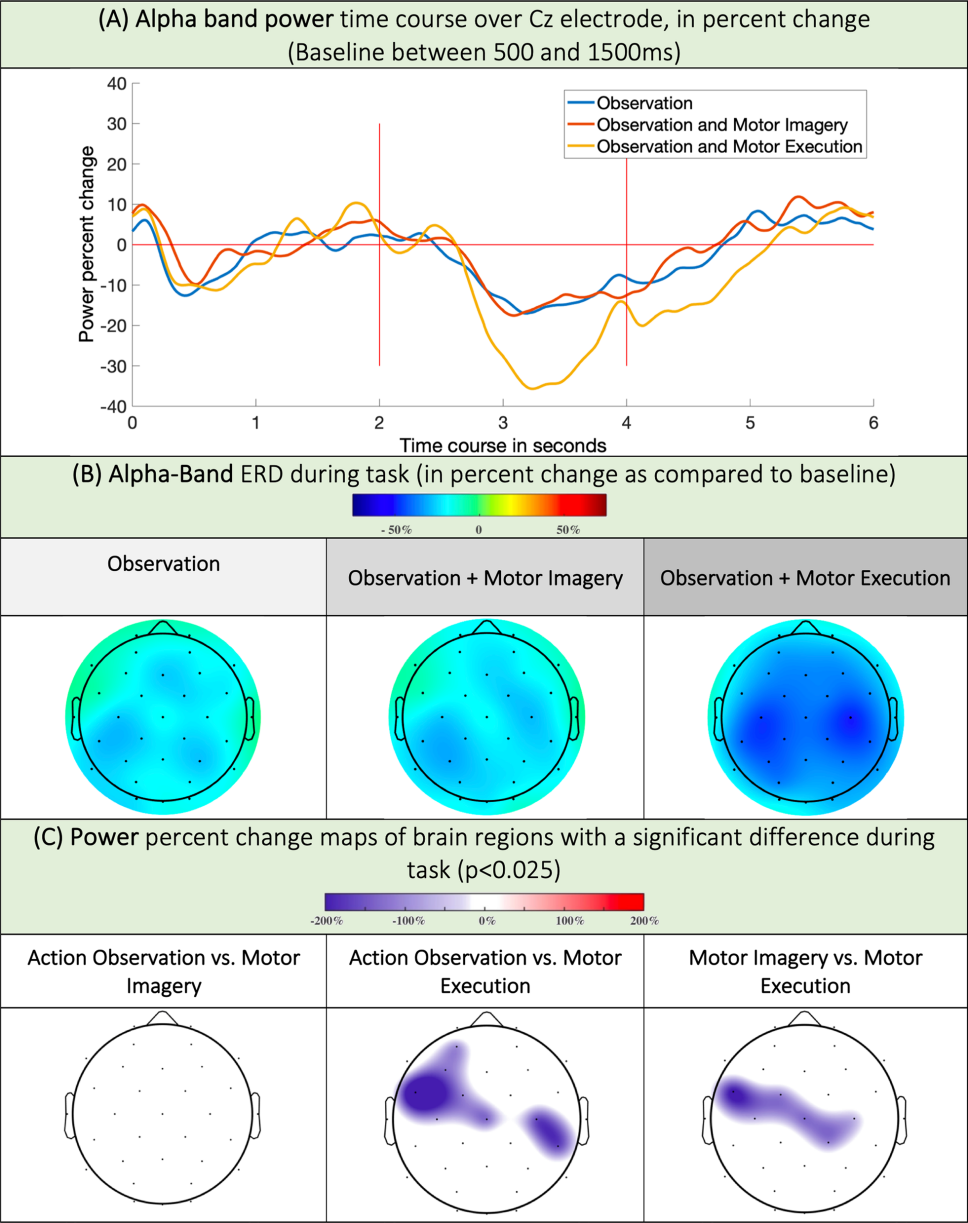
**A** Alpha band power time course, expressed as the percent change relative to the baseline (500-1500ms) band power in all conditions. **B** ERD maps in the alpha band during movement in the O, OI and OM conditions. **C** ERD relative magnitude in regions with statistically relevant changes in the alpha band. A blue color means the ERD was stronger in condition 2 versus condition 1 (i.e.: stronger ERD in Motor Execution than in Action Observation over Cz)

**Fig. 4:**
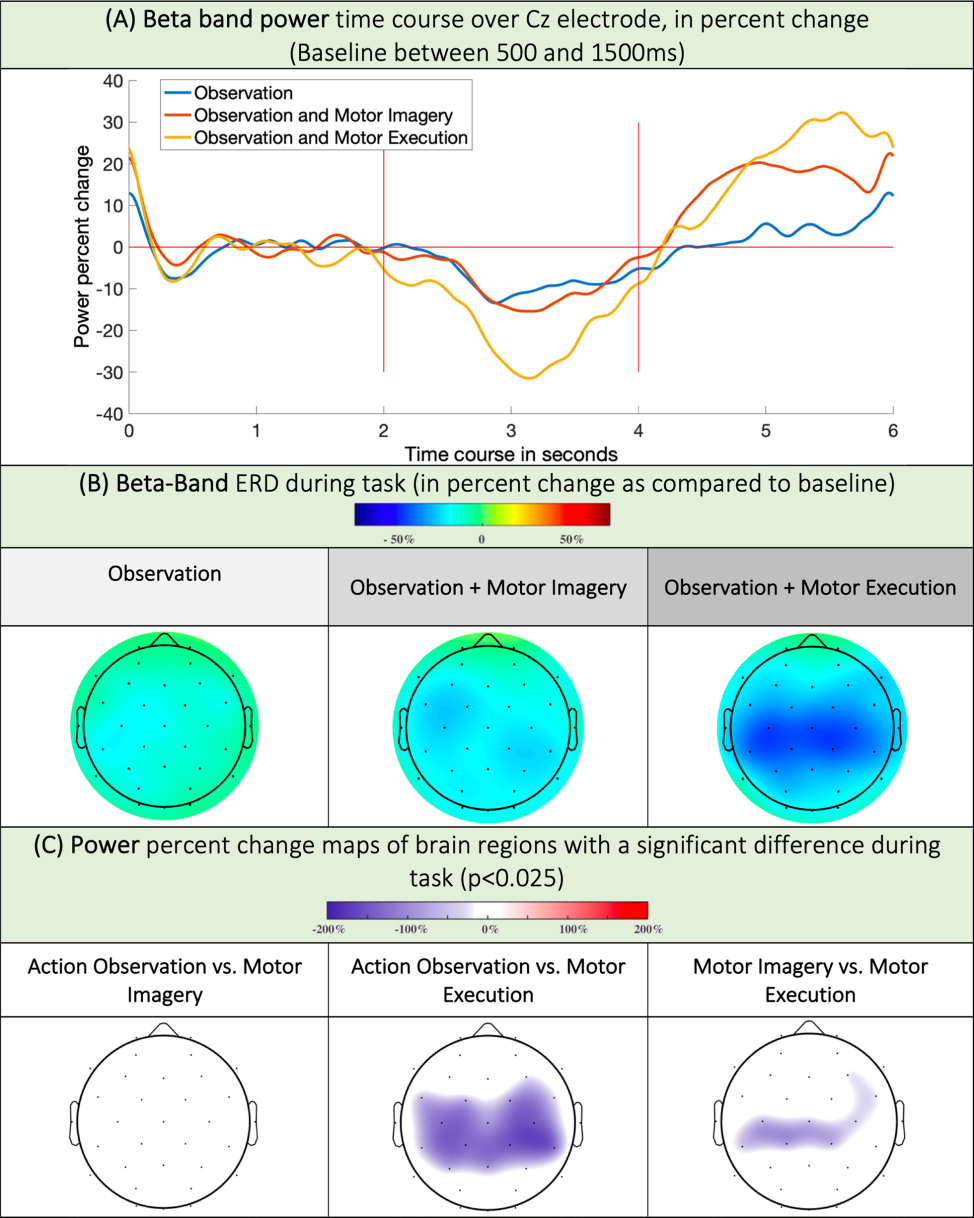
**A** Beta band power time course, expressed as the percent change relative to the baseline (500-1500ms) band power in all conditions. **B** ERD maps in the beta band during movement in the O, OI and OM conditions. **C** ERD relative magnitude in regions with statistically relevant changes in the beta band. A blue color means the ERD was stronger in condition 2 versus condition 1 (i.e.: stronger ERD in Motor Execution than in Action Observation over Cz)

**Fig. 5:**
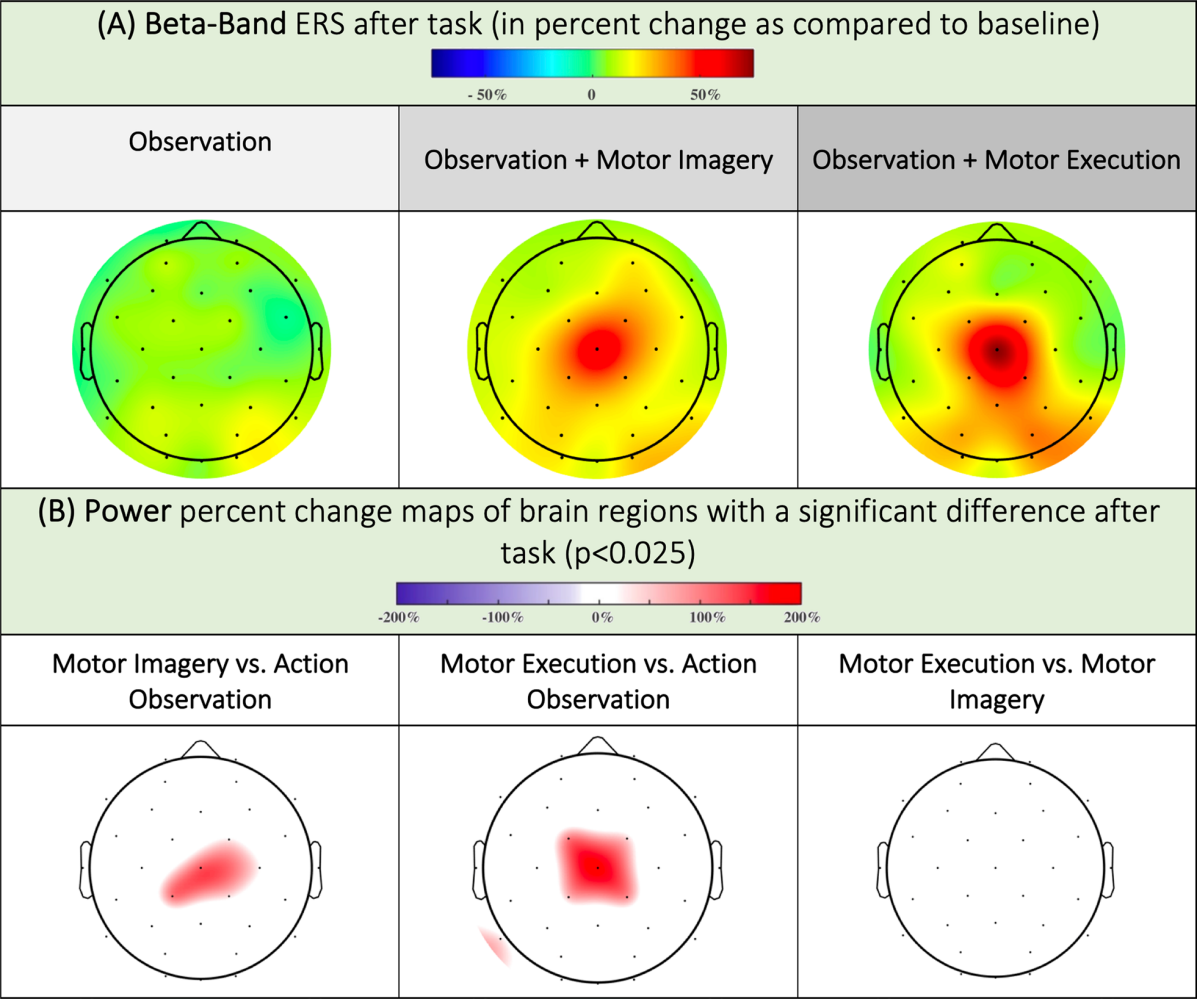
**A** ERS maps in the beta band during movement in the O, OI and OM conditions (**B**) ERS relative magnitude in regions with statistically relevant changes in the beta band. A red color means the ERS was stronger in condition 1 versus condition 2 (Stronger ERS in Motor Execution than in Action Observation over Cz and stronger ERS in Motor Imagery than in Action Observation over Cz)

**Fig. 6:**
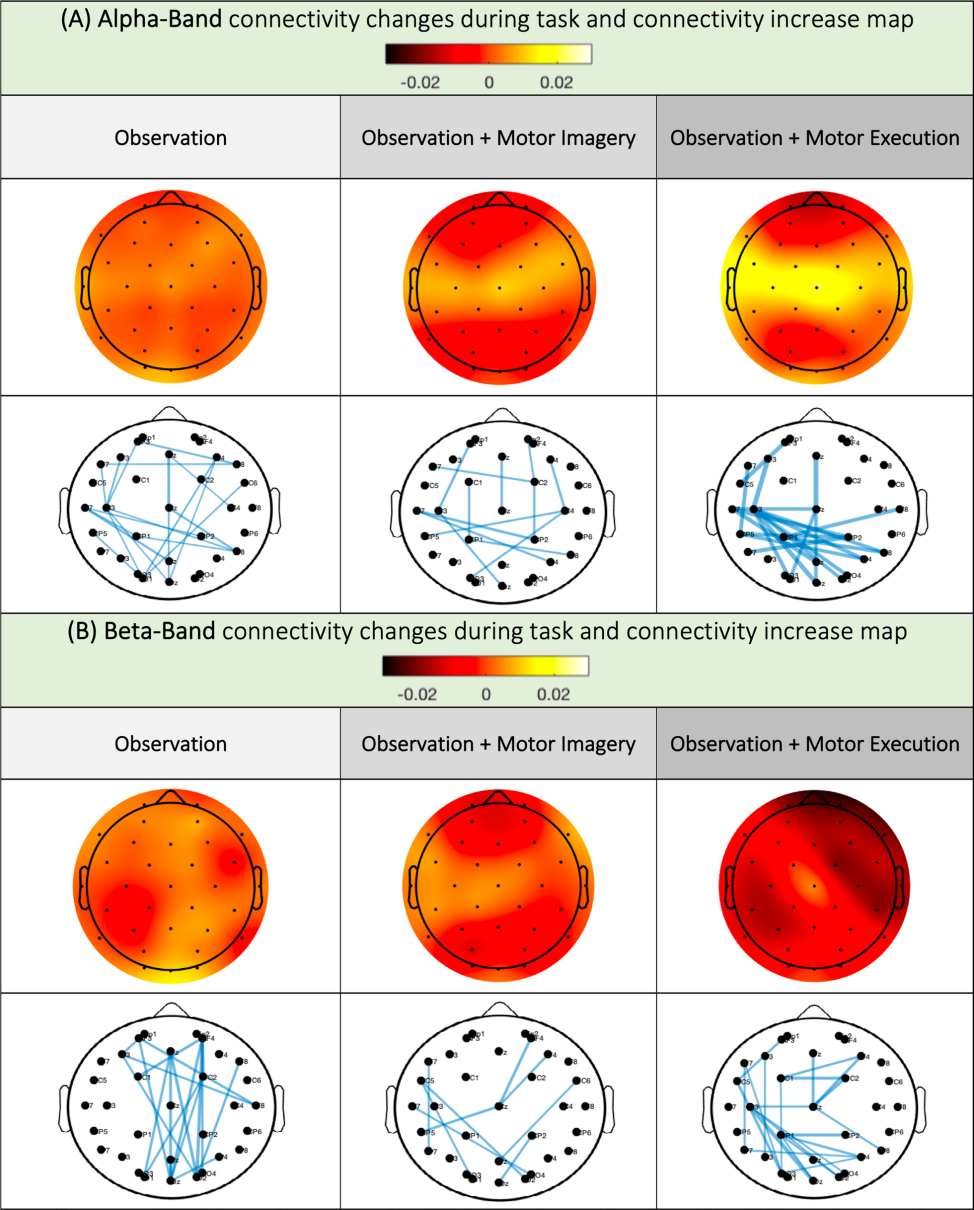
Changes in the PLV in the alpha (**A**) and beta (**B**) bands during movement compared to before movement

Time–frequency and connectivity maps are shown in Figs. 3, 4, 5, 6. Tables presenting the quantitative data from the time–frequency analyses are available in the supplementary materials (Tables 4, 5 and 6). In this section, ERD and ERS are expressed in percent change. The sensor names and positions can be found in the Table 1 of the Supplementary Materials.

### Changes in power during the task

In the alpha band during the task, we observed desynchronization in O, involving the centro-frontal, central left, and parietal electrodes. In the OI condition, desynchronization involved the fronto-central, bilateral central and parietal electrodes. In the OM condition, desynchronization was much more diffuse and powerful and was mainly centered on C3 (central left, − 22.4%—CI95 [− 29.13; − 15.67]) and C4 (central right; − 17.45%—CI95 [− 25.19; − 9.7]). Analysis of the Cz band-power time course showed that there was Cz desynchronization in all conditions, with gradations between O, OI and OM (− 8.53%—CI95 [− 12.7; − 4.37] in O; − 9.1%—CI95 [− 15.2; − 3] in OI, and − 16.21%—CI95 [− 22.41; − 10] in OM). However, in the alpha band, this Cz desynchronization remained less powerful than C3 desynchronization in all conditions (stronger C3 desynchronization).

Statistical analysis revealed no differences between the O and OI maps but confirmed that desynchronization was more powerful in the OM condition above the central and precentral electrodes than in the O and OI conditions (Fig. 3). Additional data can be found in tables 2 and 4 of Supplementary Materials.

In the beta band, in the O condition, the desynchronization was very weak, mainly over left central and the left parietal cortex (for C3 − 10.81%; CI95 [− 16.6; − 5.03], for CP5 − 10.68%; CI95 [− 17.33; − 4.02]). In the OI condition, the desynchronization recruited bilateral central, precentral and parietal electrodes and appeared stronger than in the O condition (for Cz − 12.73%; CI95 [− 17.76; − 7.7] in OI, versus − 8.99%; CI95 [− 13.68; − 4.29] in O). In the OM condition, the desynchronization was mainly centered on centro-parietal electrodes and appeared much stronger than in the O and OI conditions (for exempla, for C3: − 24.8%; CI95 [− 31.37; − 18.24] in OM).

Statistically, no difference was observed between the O and OI conditions, despite visually visible differences in the time–frequency maps. However, we confirmed a more powerful desynchronization above the bilateral precentral, central, parietal, and parieto-occipital electrodes in the OM condition than in the O condition. Desynchronization was also greater in the OM than in the OI, but only above the central and parietal electrodes but not in the precentral electrodes (Fig. 4). Additional data can be found in tables 3 and 5 of Supplementary Materials.

### Changes in power after the task

After the execution of the task, we observed a powerful rebound in the OI and OM conditions. The statistical analysis confirmed this result, with a significant difference centered on the Cz electrode (vertex) in the OI and OM conditions compared to O (for Cz: 31.87%; CI95 [18.84; 44.9] in OM versus 3.51; CI95 [6.5; 0.53] in O). There was also a visible beta rebound in the OM above the parieto-occipital electrodes (PO3: 17.59%; CI95 [10.1; 25.08], PO4: 15.27%; CI95 [9.1; 21.44]). We found no difference in rebound intensity between OIs and OMs (Fig. 5) according to the Bonferroni correction. However, without Bonferroni correction, the difference was significant, with a stronger ERS in the OM than in the OI over the Cz (p < 0.025). The time course of the rebound in the beta band is shown in Fig. 4A. Additional data can be found in tables 3 and 6 of Supplementary Materials.

### Changes in connectivity during the task

In the alpha band, we observed an increase in connectivity strength compared to connectivity before the task in O, OI and OM, above the C3, Cz, and C4 electrodes, with the greatest increase in connectivity occurring during OM conditions. Judging from the links maps, in all 3 conditions, there seemed to be strong left centro-parieto-occipital links.

In the beta band, we observed increased connectivity in the occipital regions in O and no clear change in connectivity above C3, Cz or C4. In the OI, we observed a clear increase in connectivity at Cz and C3. In the OM, there was a clear increase in connectivity above the Cz electrode, with a decrease in connectivity in the right prefrontal and left parietal regions. Interestingly, in the O condition, the links’ map showed an increase in fronto-occipital links, while in the OI and OM conditions, we again found a strong left-central-parietal-occipital link (Fig. 6).

For alpha and beta band connectivity, statistical analysis did not reveal any differences, probably because of high inter-subjects’ variability and very tenuous connectivity variations (Supplementary Materials, Table 7A and Table 8A).

### Connectivity changes after task

**Fig. 7:**
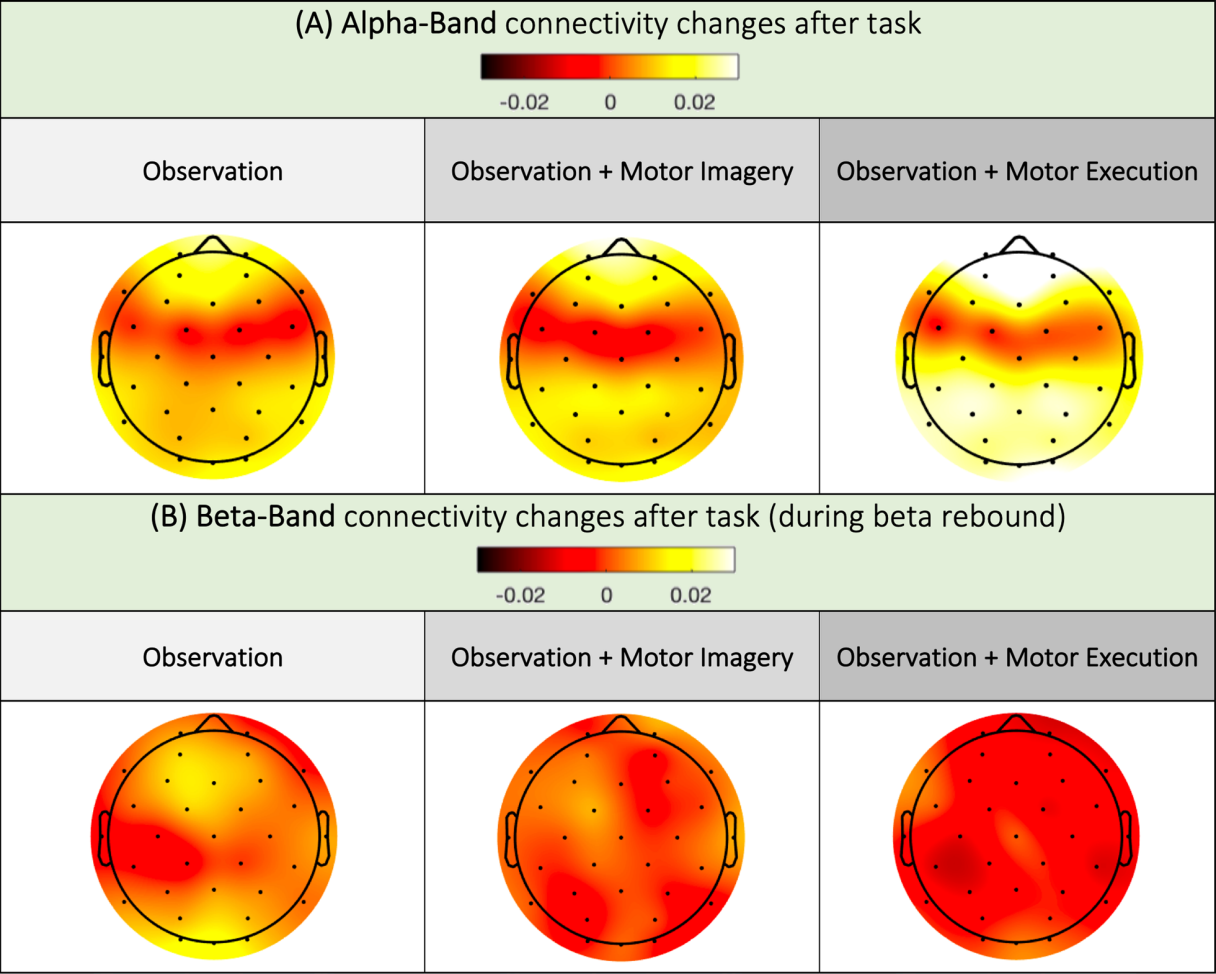
Changes in the PLV in the alpha (**A**) and beta (**B**) bands after movement compared to before movement

After the task, compared to the pre-task connectivity, we found an increase in the alpha band connectivity in the frontal, parietal and occipital areas with a gradation between O, OI and OM. The connectivity was unchanged in the central and precentral areas. Beta band connectivity after the task showed an increase in connectivity in the prefrontal and occipital areas in O. In OI and OM, the connectivity was unchanged, even above the Cz electrode (Fig. 7). The statistical analysis showed no difference between conditions. Additional data is provided in Supplementary Materials (Table 7B and Table 8B).

## Discussion

In the course of this work, for the first time, to our knowledge, we studied motor rhythms via electroencephalography during video feedback therapy of the lower limb. During the task, in the alpha band, we found a comparable ERD in O and OI over precentral, central and parietal electrodes. In the OM, the ERD involved the same regions but was stronger over central electrodes. In the beta band, there was a gradation of ERD intensity in O, OI and OM over central electrodes. After the task, the ERS (beta rebound) changes were weak during the O task but were strong in the OI and OM (Cz) groups, with no differences between the OI and OM groups.

### Recruitment of the mirror neuron system

In our study, in the O condition, we found topographies similar to those in the literature, with bilateral central and parietal desynchronization [37]. However, we observed no difference between the O and OI conditions, particularly in the parietal regions, while other works found greater alpha desynchronization in an observation task than during imagination alone [37]. Moreover, in the OM condition, the topography of desynchronization was bilateral and comparable to that in the O and OI conditions but with stronger desynchronization in the precentral and central regions; however, in a high-density EEG study, alpha desynchronization during a motor task without visual feedback led to centro-parietal recruitment only contralateral to movement [38]. These differences are probably due to the addition of an action observation task to the motor imagery and execution task. This phenomenon has already been shown in fMRI data of upper limb movements, where the addition of action observation to a motor task results in bilateral centro-parieto-occipital recruitment, whereas the motor task alone was much more lateralized [39]. We assumed that this phenomenon was linked to the recruitment of mirror neurons. Indeed, above the sensory-motor regions, alpha band desynchronization is linked to Mu desynchronization [40]. The Mu rhythm is a well-known EEG rhythm containing two independent components, one in the alpha band and one in the beta band, encoding different parameters related to motricity [23]. In the alpha band, Mu desynchronization is generally considered to indicate the activity of the mirror neuron system (MNS, 21); this activity is present not only during action observation, motor imagery, and motor execution but also in other more complex tasks recruiting mirror neurons, notably in social cognition [41]. Mu rhythm is used as a biomarker of MNS recruitment and is generated around the centro-parietal regions [42]. The observation of bilateral, centro-parietal Mu desynchronization in our O, OI and OM conditions suggested that the observation of action in video therapy in the lower limb results in recruitment of the mirror neuron system, with activation of a bilateral centro-parietal network, which we also observe in connectivity. This recruitment of the mirror neuron system has been described in mirror therapy, notably for the upper limb in healthy subjects but also for stroke patients [11, 43, 44]. For the lower limb VOT, MNS recruitment has not been documented to our knowledge.

In the OM condition, we observed significant desynchronization in the precentral and central regions compared with the O and OI conditions. It is difficult to conclude whether this greater desynchronization reflects an increase in the activity of mirror neurons or whether it is linked to other phenomena involved in motor planning or execution. Indeed, during a movement, the Mu rhythm is involved in the integration of the movement’s somatosensory parameters [23], which may enhance desynchronization in the OM task. Similarly, although there are many similarities between the observation, imagery and motor execution networks, we know that there are also some differences since, according to fMRI, only action execution systematically recruits the primary motor cortex [46]. Therefore, although it is logically expected that motor execution leads to desynchronizations of greater intensity than motor observation or imagery through greater cortical recruitment, we must remain cautious about the precise interpretation of the neurophysiological mechanism at the source of this greater alpha desynchronization in OM.

One of the pitfalls of interpreting Mu rhythms is contamination by alpha occipital activity during signal analysis, which is present in the same frequency band [41]. In this work, we did not observe desynchronization in the occipital regions, probably because the subjects were constantly focusing their visual attention on the screen. Additionally, desynchronizations seem to emerge strictly from the central and parietal regions, making the hypothesis of contamination of the observed centro-parietal desynchronization by the alpha-occipital less plausible.

### Modulation of beta activity according to the motor task

In O, we observed a weak, left parietal beta ERD. In the OI, the beta ERD was bilateral and centroparietal, and in the OM, the beta ERD was intense in the bilateral centroparietal regions. In O and OI patients, statistical analysis revealed no difference in the intensity of desynchronization. Interestingly, in OM, there was an increase in desynchronization in the motor, premotor and parietal regions compared to O. A comparison of OM and OI showed only an increase in desynchronization with respect to the central and parietal regions in favour of OM.

Beta ERD corresponds to disinhibition of somato-motor neuronal populations [23]; for example, there is a correlation between motor response intensity and desynchronization strength in stroke patient populations [24]. For lower limb movements, the beta ERD is classically localized on the vertex opposite to the moving limb [38]. Thus, since the subjects are passive and do not perform any motor planning or motor execution tasks, we did not find any clear desynchronization in O. Additionally, in the OM condition, as the subjects performed a motor task, they recruited the premotor (motor planning) and parietal (integration of proprioceptive feedback) regions, where beta desynchronization was significantly increased compared to that in the O condition. Conversely, the OI and OM comparisons revealed differences only in centro-parietal regions and not in prefrontal regions. This is probably because OIs and OMs need to develop a motor pattern (premotor cortex), but they differ in the recruitment of the primary motor cortex and parietal regions, which are much stronger in OMs (execution of task and integration of proprioceptive feedback).

Analysis of connectivity during action also reflected this gradation between the O, OI and OM conditions, with a gradation in the strength of connectivity in relation to the Cz between conditions. When analyzing connectivity links, we observed in O a fronto-occipital network, probably linked to the activation of an action observation network, whereas in OI and OM, the recruitment is mainly centro-parietal contralateral to movement and may be linked to an action planning or execution network. However, one must remain cautious about these descriptive connectivity results since no statistical difference was found between conditions.

### The need for a motor plan for post-movement validation

In our study, we observed a beta rebound emerging from the vertex, with a topography different from that of beta and alpha desynchronization; this rebound was absent in O but present in OI and OM. Beta rebounding corresponds to hypersynchrony in the beta band following movement [33, 48]. It originates in the precentral gyrus, more precisely, in the motor cortex [49, 50]. Initially, described as participating in the maintenance of an idling state in sensorimotor regions, its interpretation has been broadened [51]. It appears that beta rebound is modulated by motor validation phenomena and temporal integration of somatosensory and motor parameters [26]. For example, the observation of an erroneous movement can modulate beta rebound [25], as can the introduction of errors in a motor task [52]. It is possible that this modulation of beta rebound emerges following the detection of a mismatch between the forward model and the sensory afferents, allowing an update of the motor pattern [53]. Our results seem to confirm this hypothesis, as we observed one desynchronization in OI and OM but no desynchronization in O. This finding confirms that the vision of a movement, even from a 1st-person point of view, will trigger a significant beta rebound only if it is perceived as feedback for a motor pattern, which is either executed (OM) or simulated (OI).

No significant difference in rebound intensity was observed between the OI and OM groups, although there was a proprioceptive/visual mismatch in the OI group (the leg was motionless during the video in the OI group). Negative proprioceptive feedback is known to negatively modulate beta rebound [51]. Providing correct visual feedback in video therapy could therefore minimize the effects of incorrect proprioceptive feedback on rebound formation. This result is of particular interest in neurological rehabilitation, where beta rebound is known to be a marker for monitoring neurological recovery [24]. However, to test this hypothesis, it would be interesting to study beta rebound in patients with cerebellar stroke (alteration of the forward model [54, 55]) and patients with proprioceptive disorders.

Interestingly, in terms of connectivity after the task, we did not observe an increase in connectivity in the beta band over the Cz, suggesting a topographically localized phenomenon. However, we observed an increase in connectivity in the frontal, parietal and occipital regions in the alpha band, with a gradation between the O, OI and OM tasks. This increase in connectivity was associated with a decrease in prefrontal connectivity. To our knowledge, this change in connectivity has never been described previously. We know that there are differences in low-beta and high-beta band function during beta rebound [53], with involvement of the frontal and parietal cortexes in addition to the motor regions [56]. However, our observations were in the alpha band and may be indicative of another mechanism involved. Additionally, PLV connectivity is sensitive to volume conduction, making topographical analysis of such broad phenomena less robust. We must therefore remain cautious when interpreting these observations.

### From healthy subjects to neurological patients

As this study was carried out on healthy subjects, we must remain cautious regarding the transposition of these results to a pathological population, with extremely different functional cortical dynamics and brain rhythms [57]. However, some general conclusions can be formulated.

Firstly, this work demonstrates a gradation of engagement between the tasks (O, OI, OM), that enables personalization of the therapy offered to patients according to their level of recovery. Indeed, we demonstrate that passive observation in lower limb video feedback therapy leads to sensori-motor cortical recruitment. Although weak, this activation could be of interest in the very early phases of neurological rehabilitation, for example to tackle maladaptive plasticity as demonstrated for upper limb video feedback therapy [58]. However, it seems necessary to work with motor imagery or motor execution tasks as soon as possible, to maximize cortical recruitment and to trigger motor validation phenomena such as beta rebound, which are absent in passive observation task.

Also, this work provides a physiological basis for understanding the specific effects of visual feedback. Indeed, many rehabilitation studies focus on mirror therapy, which combines two distinct tasks: (1) a systematic movement of the patient’s healthy hand or foot, and (2) an observation of the visual feedback on the mirror. Yet these two tasks have distinct neurological effects, whose interpretation often gets mixed up. Indeed, for the upper limb a bimanual movement may lead to bimanual facilitation [59], with an increase of motor cortex excitability [60] and a modulation of EEG rhythms and connectivity [61], which can bias the understanding of the specific effects of the visual feedback. Yet, the understanding of these specific effects is crucial to the personalization of the therapies. For example, we don’t know how the visual feedback is integrated in patients with neuro-visual disorders, or if proprioceptive disorders may conflict with correct the visual feedback, and thus decrease the sensori-motor cortex recruitment.

We also have few points of comparison between lower and upper limb video feedback therapy since most of the studies on visual feedback focus on upper limb rehabilitation. The main EEG rhythms dynamics (ERD, ERS) seem to be generally the same, with a different topography above for motor areas, with a gradation between O, OI, and OM cortical recruitment between tasks, and enhanced recruitment in action observation as compared to motor imagery alone [37]. Yet, many questions hypothesis demonstrated for upper limb rehabilitation remain to be tested for lower limb rehabilitation. For example, for upper limb rehabilitation, in an action observation and motor imagery condition (similar to our OI), it appears that the alpha band ERD is enhanced by the vision of own hand movement, as compared to a non-subjective movement [19], especially in a first person perspective [45]. Considering that action observation facilitates motor cortical activity after stroke [12], we believe that lower limb rehabilitation with action observation should always try to implement a first person subjective visual feedback, in order to maximize the cortical recruitment. This hypothesis remains however to be tested.

Our next step will be to study the specific effects of the visual feedback for stroke patients, regarding their lesion topography.

### Limitations

There are several limitations to this work. First, we chose to add a two second interval between the end of the video and the beginning of the next epoch, in order to monitor the beta rebound. However, this interval appeared to be short since the beta rebound had not entirely returned to baseline at the start of the next epoch. For future work we will consider putting at least three seconds between the end of a video and the beginning of the next epoch.

A longer time at the beginning of the epochs should also be considered. Indeed, we observed in all the time–frequency maps an artifact at the beginning of the signal, that may have been caused by an event related potential due to a slight saccade in the video loop, maximal over occipital brain regions. Our baseline may also have been contaminated by some motor-preparation rhythms. Statistical comparison was performed between time–frequency maps with different baselines (500–1500 ms baseline versus 700–1200 ms baseline) and found no difference. Even if this did not change the overall significancy of our results, further studies should include at least three seconds of pause at the beginning of each epoch.

Finally, for connectivity we chose to perform a PLV analysis, which can be sensitive to volume conduction effects. We tried to mitigate these volume conduction effects by using a surface Laplacian filter [34]. However, PLI (Phase Lag Index) and wPLI (Weighted Phase Lag Index) connectivity analysis, which are insensitive to volume conduction artifacts, showed no interpretable results. Though this work presents original connectivity data during lower limb visual-feedback therapy tasks, interpretation of these connectivity changes between conditions must be cautious, especially considering the absence of statistically significant results. A specific study of connectivity changes using a high-density EEG headset and a source level analysis could prove interesting.

## Conclusion

In this work, we investigated for the first time the EEG correlates of video feedback therapy to the lower limb under three conditions: action observation alone, action observation with motor imaging, and action observation with motor execution.

We observed bilateral centro-parietal alpha desynchronization in all conditions, corresponding to the activation of the mirror neuron system, with strong sensory-motor recruitment in motor execution. The study of beta desynchronization showed a gradation according to motor task O, OI and OM, with recruitment of the premotor cortex in the OI and OM and of the motor cortex and parietal regions in the OM. Finally, the study of beta rebounds highlights the need to add motor intention to action observation to trigger motor validation mechanisms.

This study provides a better understanding of the neurophysiological effects of video observational therapy and supports the benefits of adding visual feedback in support of motor imagery or motor execution during rehabilitation of the lower limb.

### Supplementary Information

**Additional file 1: **Table 1: EEG sensors map. C3 electrode is placed over left sensori-motor cortex, while C4 electrode is placed over right sensori-motor cortex.

**Additional file 2: **Table 2: Power time course in the alpha band expressed in percent change as compared to baseline (500—1500ms) over C3, Cz and C4 electrodes, in all conditions. The shaded area represents the 95% confidence interval.

**Additional file 3: **Table 3: Power time course in the beta band expressed in percent change as compared to baseline (500—1500ms) over C3, Cz and C4 electrodes, in all conditions. The shaded area represents the 95% confidence interval.

**Additional file 4: **Table 4: Mean power value in the alpha band during task for all electrodes, in the three conditions, expressed in percent change as compared to baseline (500—1500 ms), with 95% confidence interval. Sensors placed above sensori-motor cortex are highlighted in grey (C3, Cz, C4).

**Additional file 5: **Table 5: Mean power value in the alpha band during task for all electrodes, in the three conditions, expressed in percent change as compared to baseline (500—1500 ms), with 95% confidence interval. Sensors placed above sensori-motor cortex are highlighted in grey (C3, Cz, C4).

**Additional file 6: **Table 6: Mean power value in the beta band after task for all electrodes, in the three conditions, expressed in percent change as compared to baseline (500—1500 ms), with 95% confidence interval. Sensors placed above sensori-motor cortex are highlighted in grey (C3, Cz, C4).

**Additional file 7: **Table 7: Alpha-band PLV changes distribution and mean between subjects during task (A) and after task (B). Mean value is represented by a grey square.

**Additional file 8: **Table 8: Beta-band PLV changes distribution and mean between subjects during task (A) and after task (B). Mean value is represented by a grey square.

**Additional file 9: **Table 9: Time frequency percent changes distribution and mean between subjects during task in the alpha band (A), during task in the beta band (B) and after task in the beta band (C). Mean value is represented by a grey square.

## Data Availability

The datasets supporting the conclusions of this article are available upon reasonable request.

## Abbreviations

EEG: Electroencephalography
ERD: Event related desynchronization
ERS: Event related synchronization
fMRI: Functional MRI
FWHM: Full width at half maximum
O: Passive action observation
OI: Action observation with motor imagery
OM: Action observation with motor execution
PLV: Phase locking value
VOT: Video observational therapy
