# Supplementary figures and images for: Neural basis of lower-limb visual feedback therapy: an EEG study in healthy subjects
Source: J Neuroeng Rehabil. 2024 Jul 8;21:114. doi: 10.1186/s12984-024-01408-8 (PMC11229246; doi:10.1186/s12984-024-01408-8)

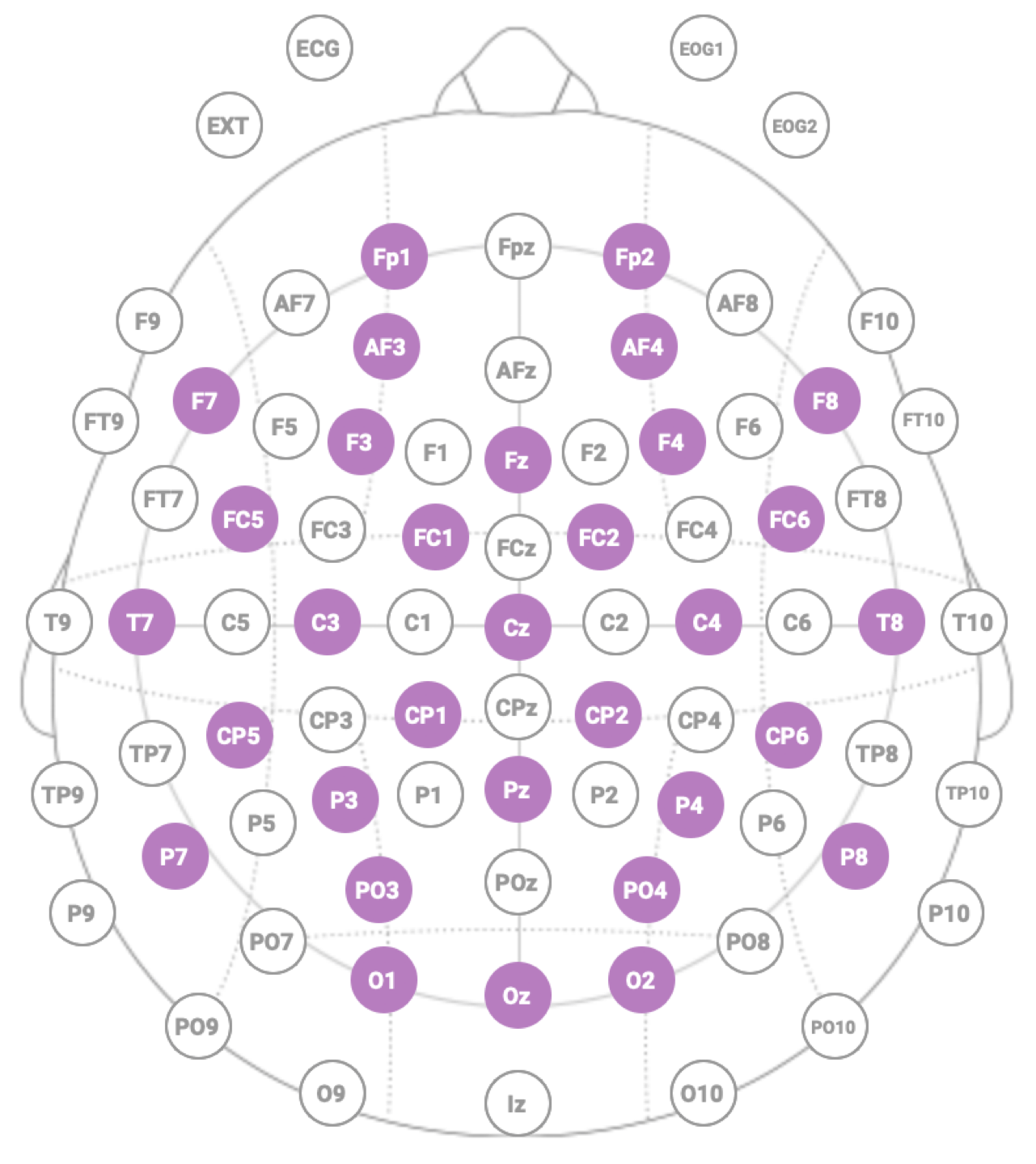

Supplement: Supplementary file 1 — Additional file 1: Table 1: EEG sensors map. C3 electrode is placed over left sensori-motor cortex, while C4 electrode is placed over right sensori-motor cortex. [file 12984_2024_1408_MOESM1_ESM.tiff]

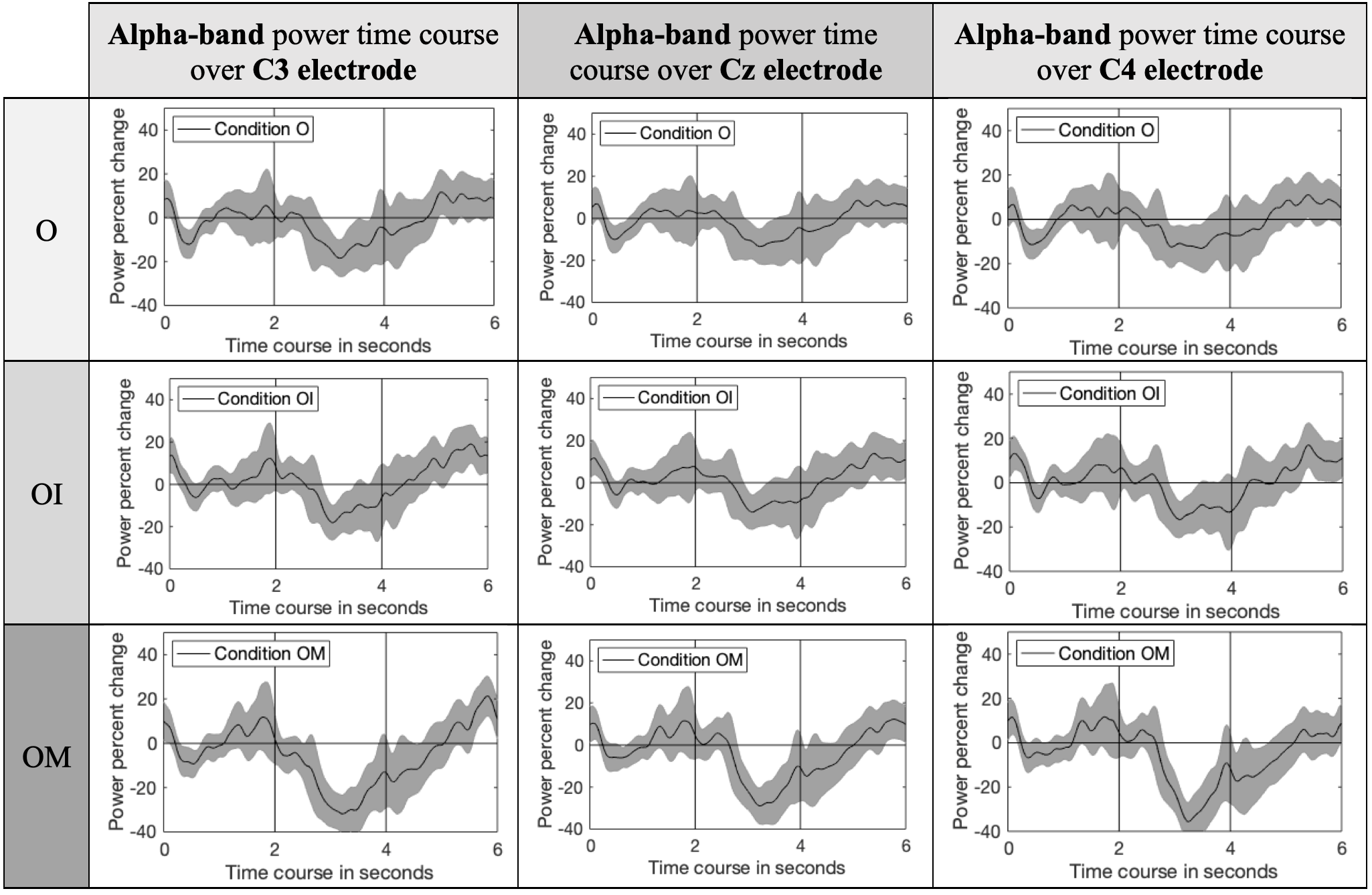

Supplement: Supplementary file 2 — Additional file 2: Table 2: Power time course in the alpha band expressed in percent change as compared to baseline (500—1500ms) over C3, Cz and C4 electrodes, in all conditions. The shaded area represents the 95% confidence interval. [file 12984_2024_1408_MOESM2_ESM.tiff]

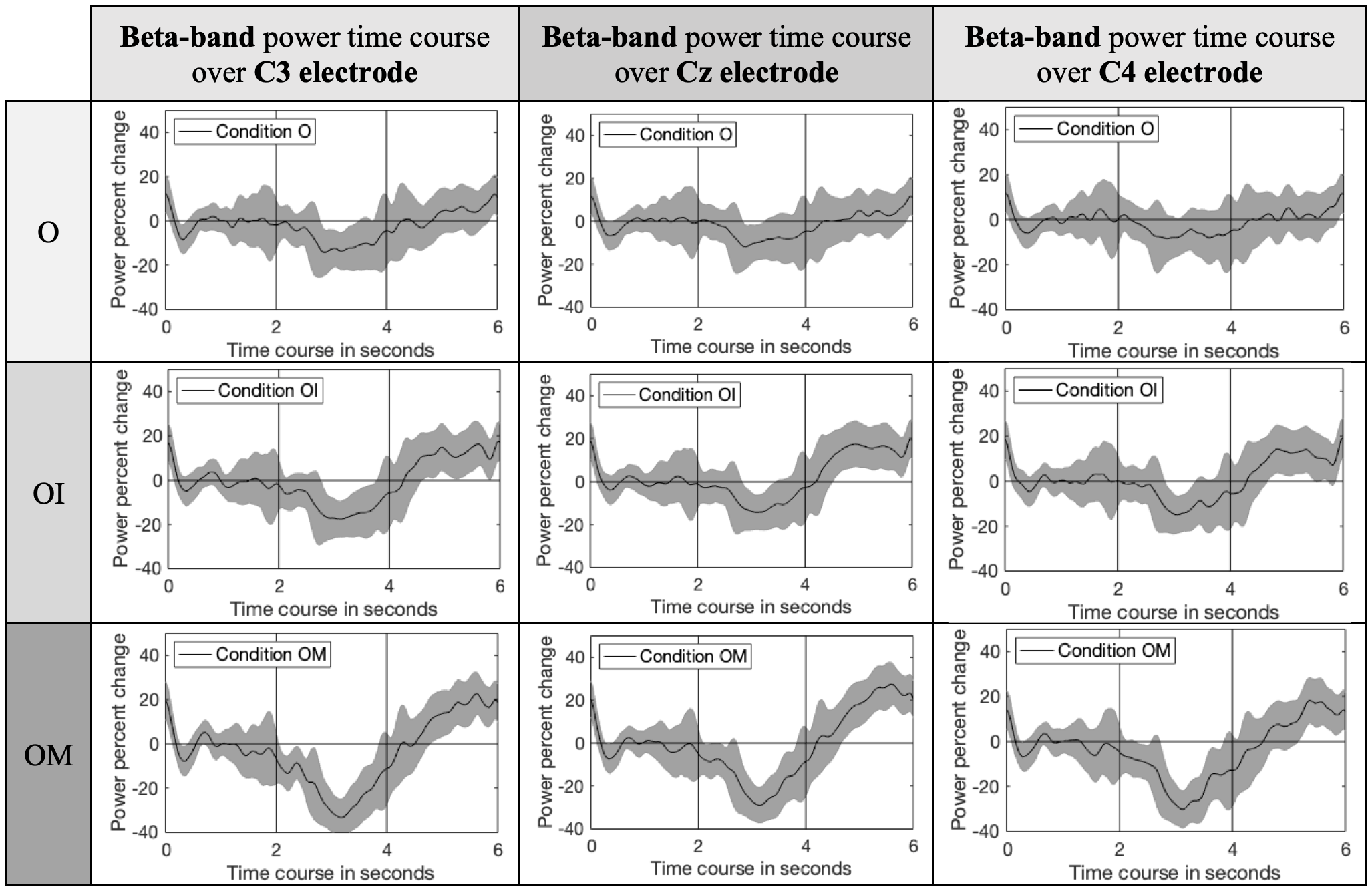

Supplement: Supplementary file 3 — Additional file 3: Table 3: Power time course in the beta band expressed in percent change as compared to baseline (500—1500ms) over C3, Cz and C4 electrodes, in all conditions. The shaded area represents the 95% confidence interval. [file 12984_2024_1408_MOESM3_ESM.tiff]

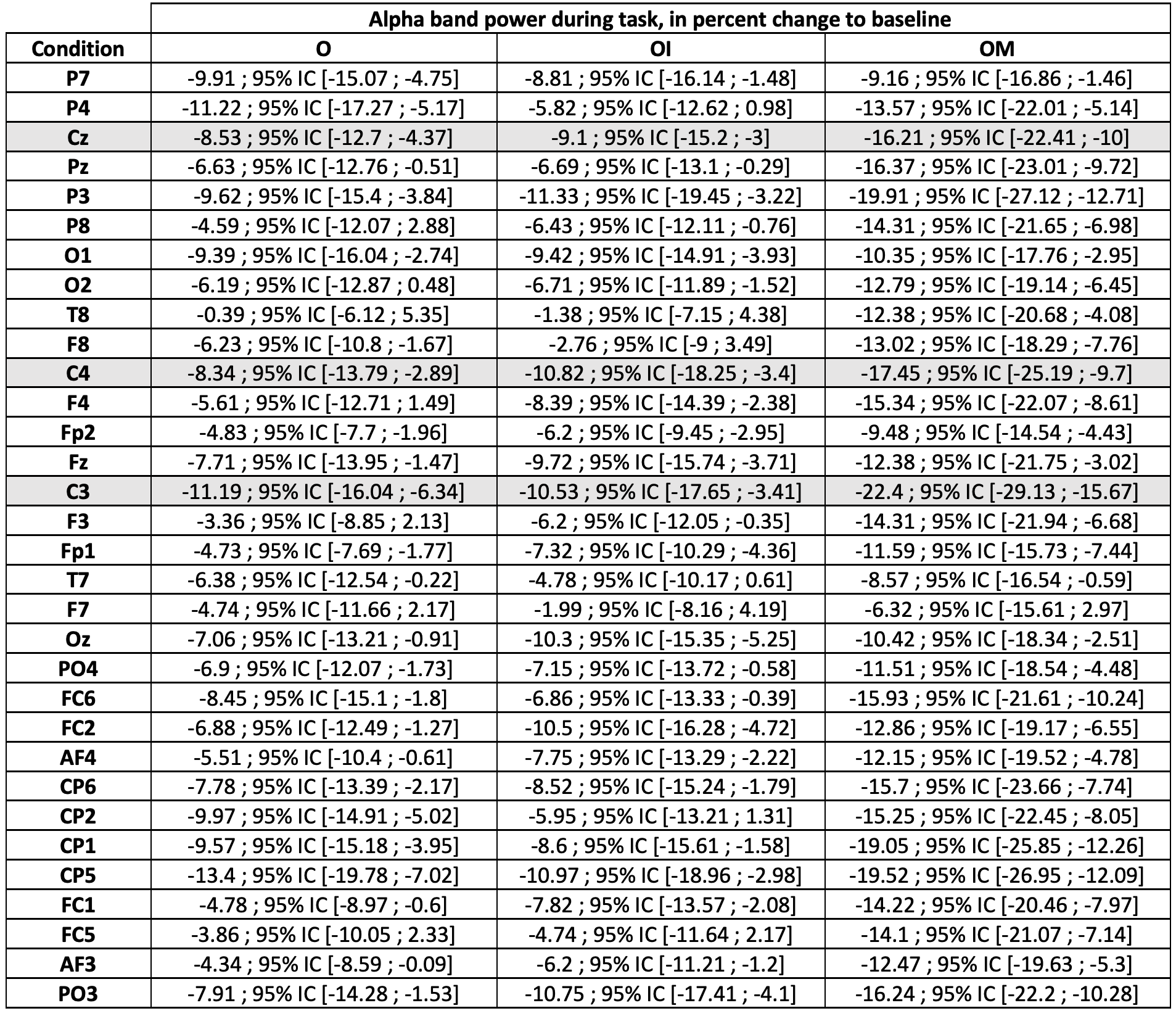

Supplement: Supplementary file 4 — Additional file 4: Table 4: Mean power value in the alpha band during task for all electrodes, in the three conditions, expressed in percent change as compared to baseline (500—1500 ms), with 95% confidence interval. Sensors placed above sensori-motor cortex are highlighted in grey (C3, Cz, C4). [file 12984_2024_1408_MOESM4_ESM.tiff]

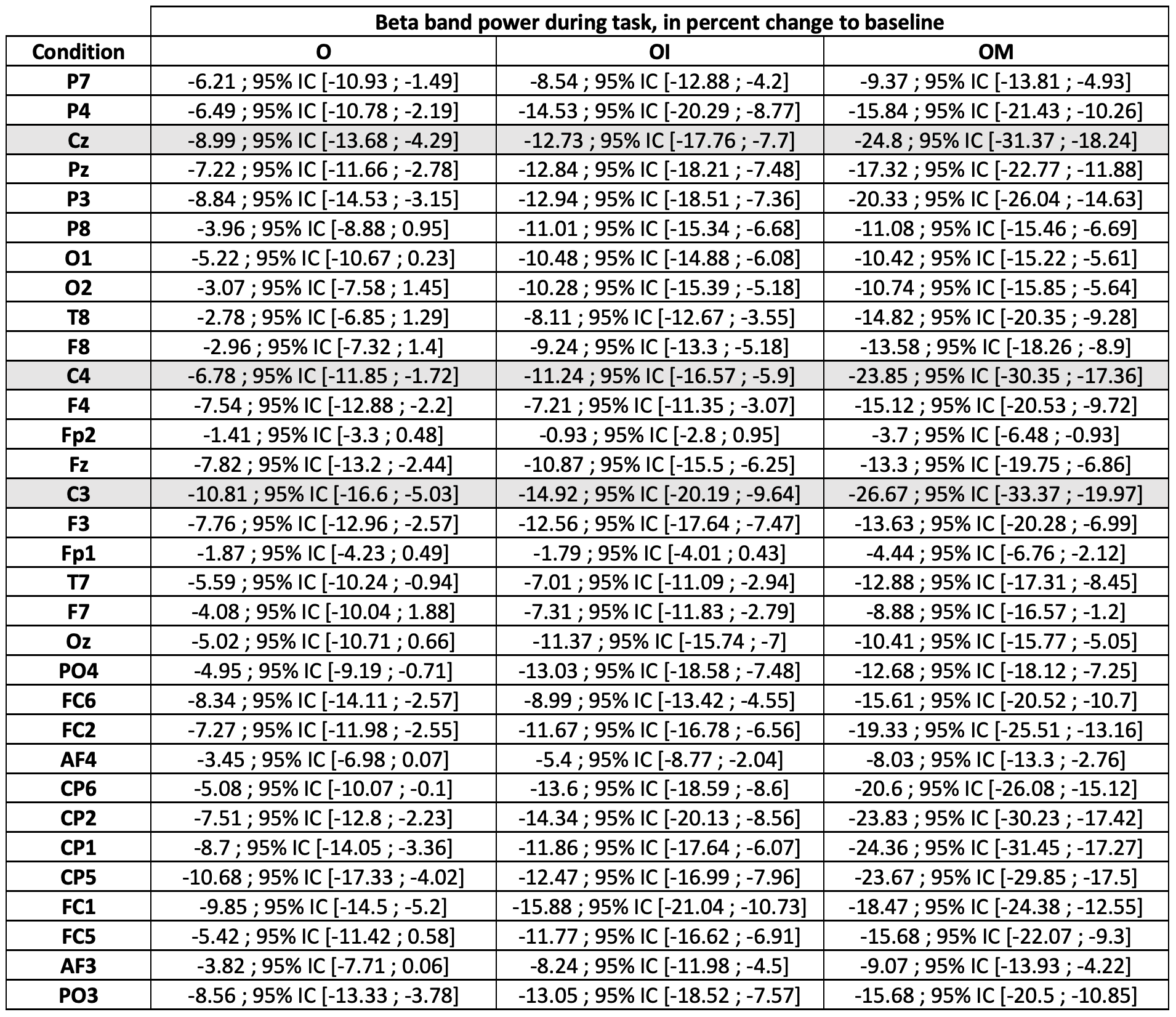

Supplement: Supplementary file 5 — Additional file 5: Table 5: Mean power value in the alpha band during task for all electrodes, in the three conditions, expressed in percent change as compared to baseline (500—1500 ms), with 95% confidence interval. Sensors placed above sensori-motor cortex are highlighted in grey (C3, Cz, C4). [file 12984_2024_1408_MOESM5_ESM.tiff]

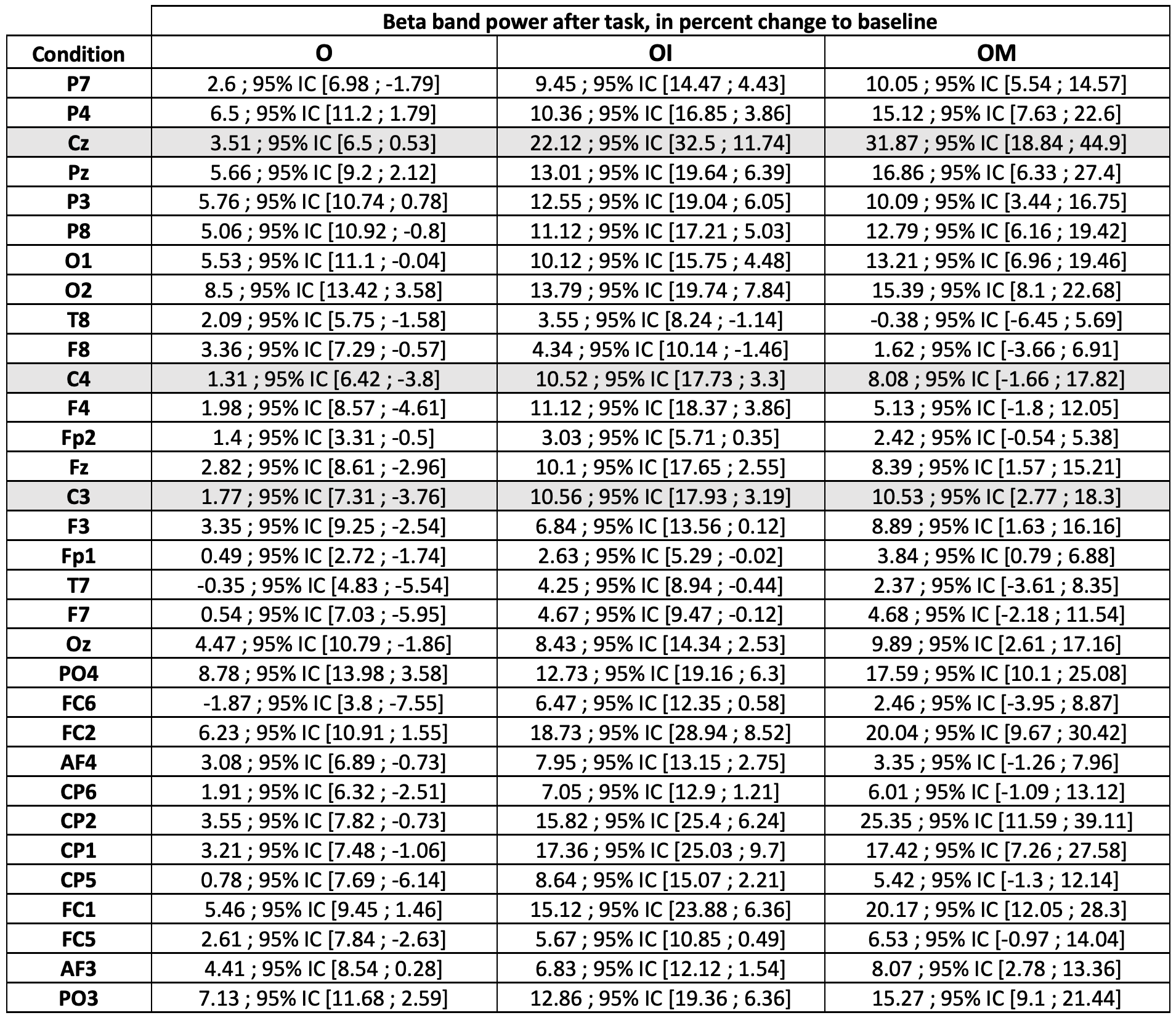

Supplement: Supplementary file 6 — Additional file 6: Table 6: Mean power value in the beta band after task for all electrodes, in the three conditions, expressed in percent change as compared to baseline (500—1500 ms), with 95% confidence interval. Sensors placed above sensori-motor cortex are highlighted in grey (C3, Cz, C4). [file 12984_2024_1408_MOESM6_ESM.tiff]

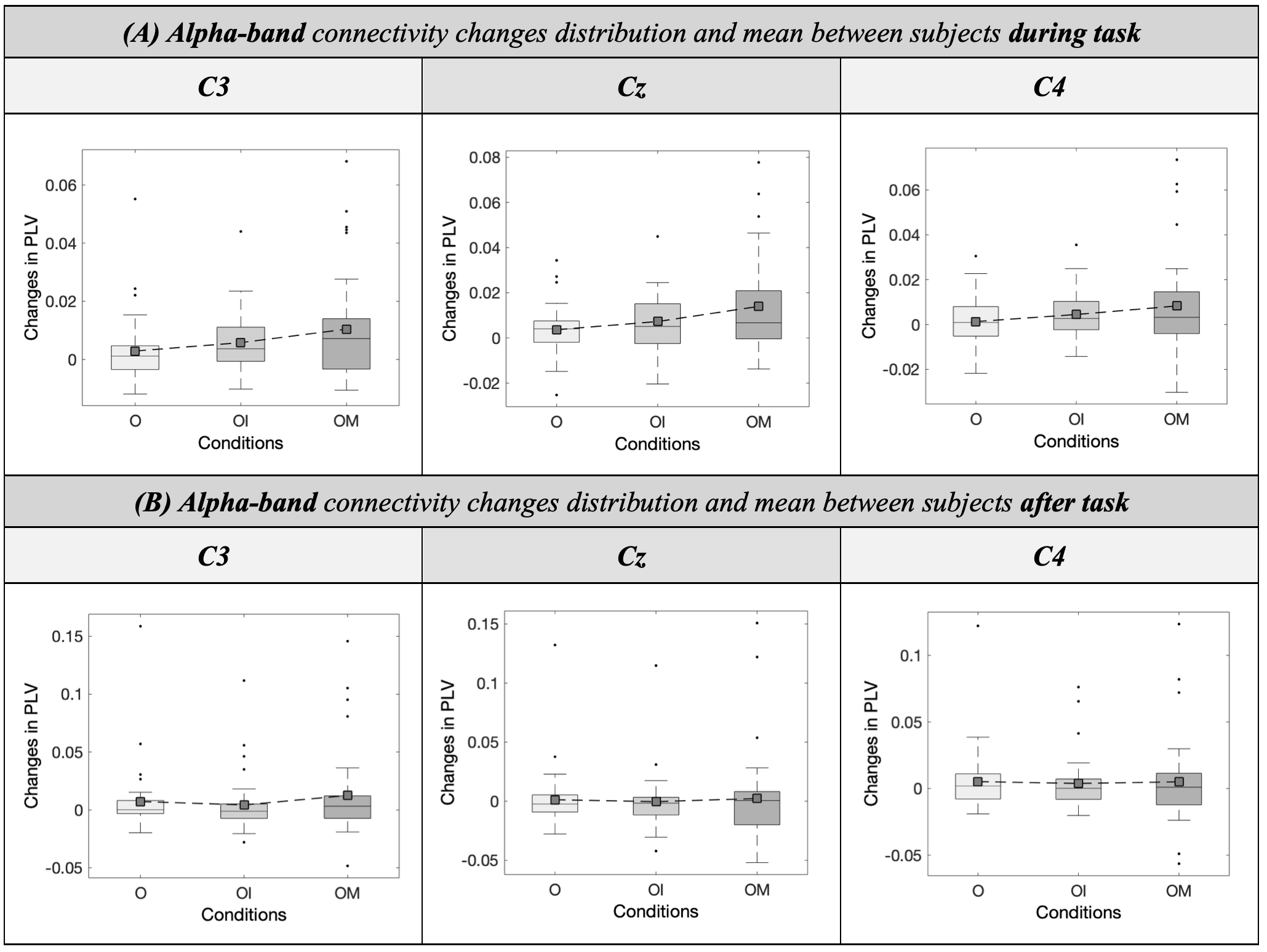

Supplement: Supplementary file 7 — Additional file 7: Table 7: Alpha-band PLV changes distribution and mean between subjects during task (A) and after task (B). Mean value is represented by a grey square. [file 12984_2024_1408_MOESM7_ESM.tiff]

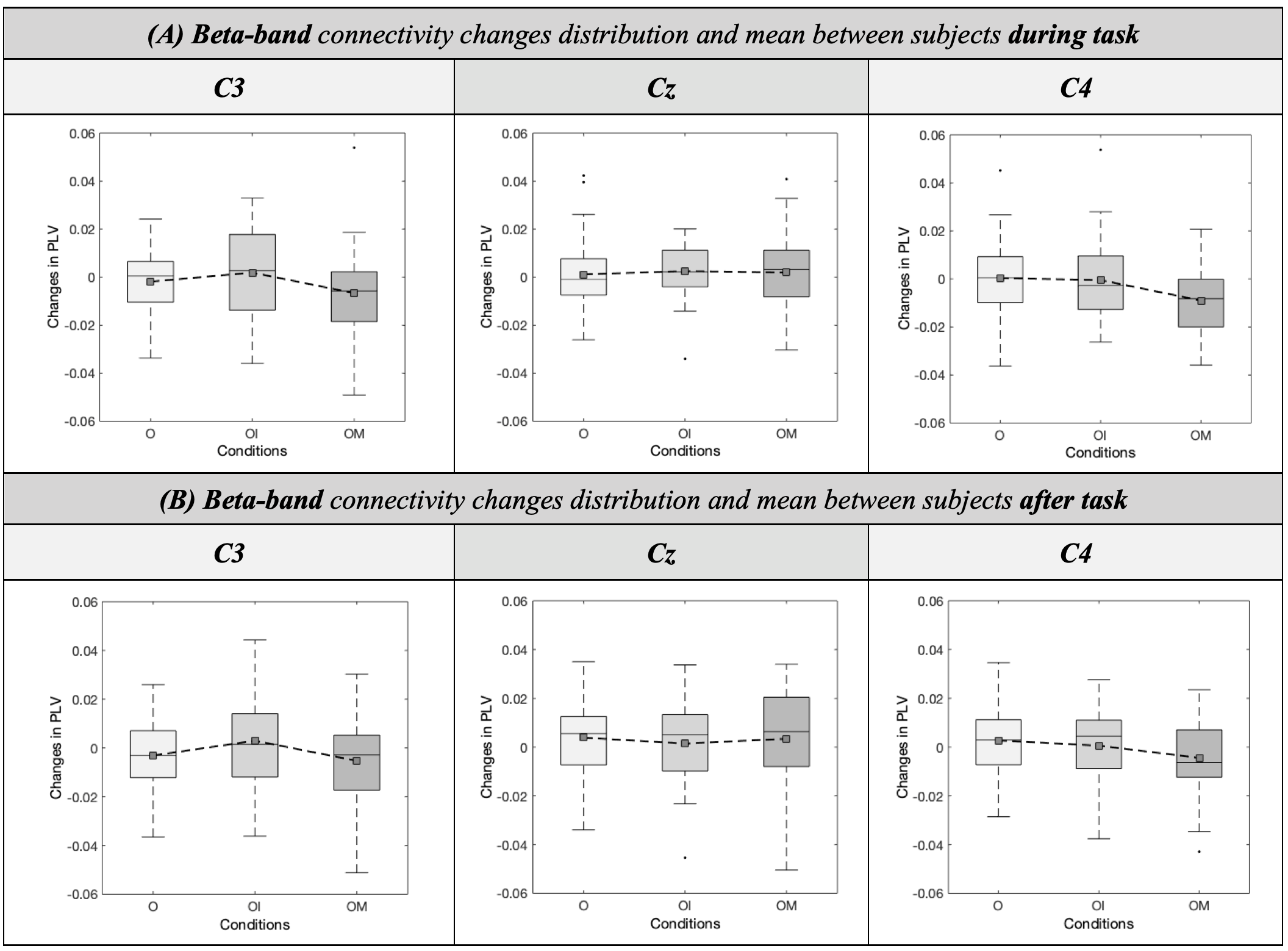

Supplement: Supplementary file 8 — Additional file 8: Table 8: Beta-band PLV changes distribution and mean between subjects during task (A) and after task (B). Mean value is represented by a grey square. [file 12984_2024_1408_MOESM8_ESM.tiff]

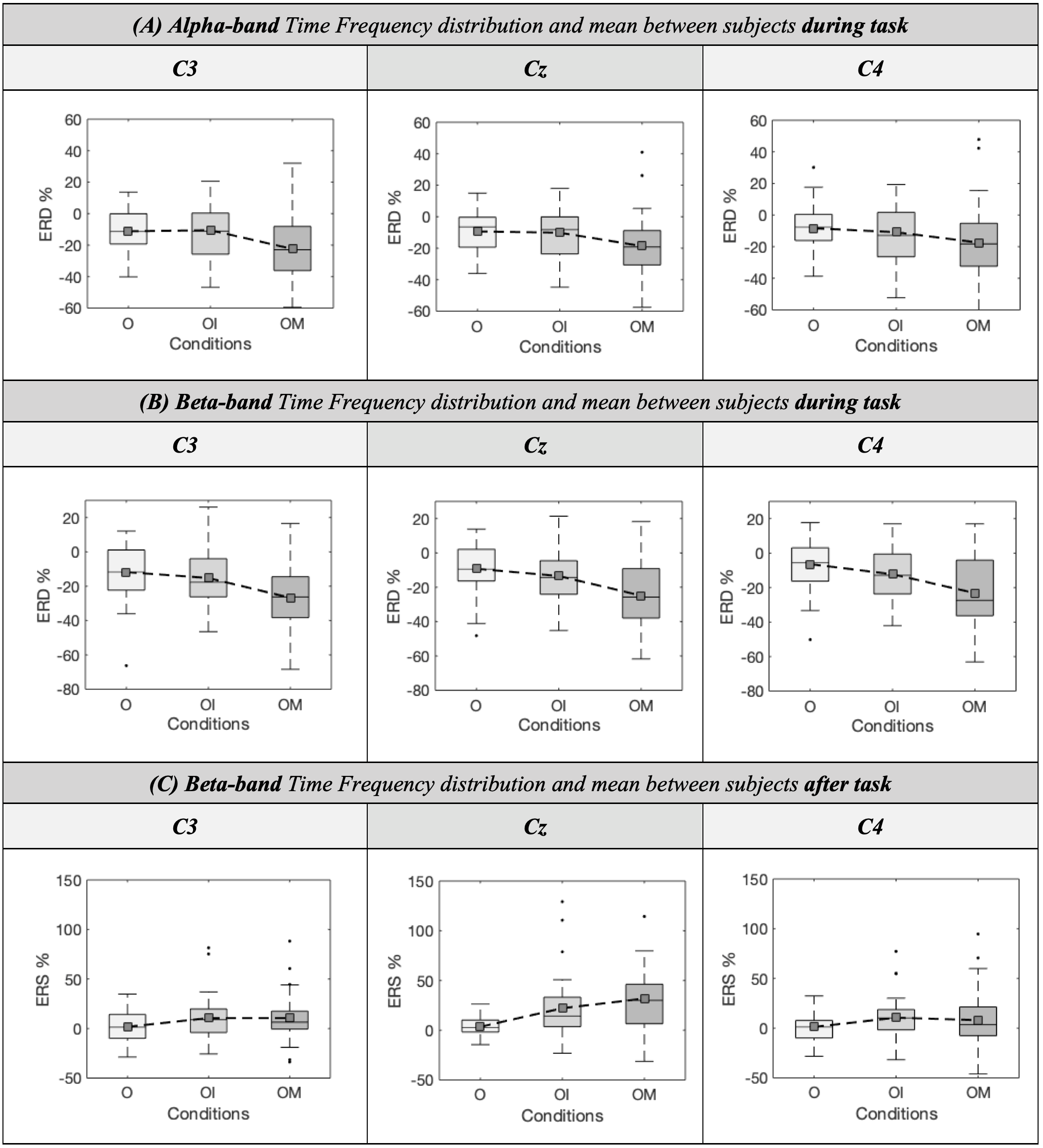

Supplement: Supplementary file 9 — Additional file 9: Table 9: Time frequency percent changes distribution and mean between subjects during task in the alpha band (A), during task in the beta band (B) and after task in the beta band (C). Mean value is represented by a grey square. [file 12984_2024_1408_MOESM9_ESM.tiff]
